# Monitoring diabetes in patients with and without rheumatoid arthritis: a Medicare study

**DOI:** 10.1186/ar3915

**Published:** 2012-07-18

**Authors:** Christie M Bartels, Jessica M Saucier, Carolyn T Thorpe, Amy JH Kind, Nancy Pandhi, Karen E Hansen, Maureen A Smith

**Affiliations:** 1 Department of Medicine, Rheumatology Section, University of Wisconsin School of Medicine and Public Health, 600 N. Highland Ave., Madison, WI 53792, USA; 2 Departments of Medicine and Dermatology, University of Wisconsin School of Medicine and Public Health, 600 N. Highland Ave., Madison, WI 53792, USA; 3 Health Services Research and Development, Veterans Affairs Pittsburgh Healthcare System, University Drive, Pittsburgh, PA 15240, USA; 4 Department of Pharmacy and Therapeutics, School of Pharmacy, University of Pittsburgh, 1140 Salk Hall, 3501 Terrace St., Pittsburgh, PA 15261, USA; 5 Department of Medicine, Geriatrics Division, University of Wisconsin School of Medicine and Public Health, 600 Highland Ave., Madison, WI 53792, USA; 6 William S. Middleton Hospital, Geriatric Research Education and Clinical Center, United States Department of Veterans Affairs, 2500 Overlook Terrace, Madison, WI 53705, USA; 7 Department of Family Medicine, University of Wisconsin School of Medicine and Public Health, 1100 Delaplaine Ct., Madison, WI 53715, USA; 8 Department of Population Health Sciences, University of Wisconsin School of Medicine and Public Health, 707 WARF Building, 610 North Walnut St., Madison, WI 53726, USA; 9 Department of Surgery, University of Wisconsin School of Medicine and Public Health, 600 Highland Ave., Madison, WI 53792, USA

## Abstract

**Introduction:**

Diabetes mellitus is a key predictor of mortality in rheumatoid arthritis (RA) patients. Both RA and diabetes increase the risk of cardiovascular disease (CVD), yet understanding of how comorbid RA impacts the receipt of guideline-based diabetes care is limited. The purpose of this study was to examine how the presence of RA affected hemoglobin A1C (A1c) and lipid measurement in older adults with diabetes.

**Methods:**

Using a retrospective cohort approach, we identified beneficiaries ≥65 years old with diabetes from a 5% random national sample of 2004 to 2005 Medicare patients (N = 256,331), then examined whether these patients had comorbid RA and whether they received guideline recommended A1c and lipid testing in 2006. Multivariate logistic regression was used to examine the effect of RA on receiving guideline recommended testing, adjusting for baseline sociodemographics, comorbidities and health care utilization.

**Results:**

Two percent of diabetes patients had comorbid RA (N = 5,572). Diabetes patients with comorbid RA were more likely than those without RA to have baseline cardiovascular disease (such as 17% more congestive heart failure), diabetes-related complications including kidney disease (19% higher), lower extremity ulcers (77% higher) and peripheral vascular disease (32% higher). In adjusted models, diabetes patients with RA were less likely to receive recommended A1c testing (odds ratio (OR) 0.84, CI 0.80 to 0.89) than those without RA, but were slightly more likely to receive lipid testing (OR 1.08, CI 1.01 to 1.16).

**Conclusions:**

In older adults with diabetes, the presence of comorbid RA predicted lower rates of A1c testing but slightly improved lipid testing. Future research should examine strategies to improve A1c testing in patients with diabetes and RA, in light of increased CVD and microvascular risks in patients with both conditions.

## Introduction

The Centers for Disease Control and Prevention estimate that >26% of adults over 65 have diabetes mellitus (DM), and reports note that diabetes is a key mortality predictor in patients with rheumatoid arthritis (RA) [1-3]. Both RA and DM increase cardiovascular disease (CVD) risk; thus, the question of how comorbid RA impacts diabetes care merits consideration, but has received little prior attention. It is generally accepted that diabetes control, monitored by American Diabetes Association recommended annual cholesterol and biannual hemoglobin A1C (A1c) testing [4], reduces macro- and microvascular complications, respectively [5-7]. At the same time, the European League Against Rheumatism (EULAR) recommends "annual cardiovascular risk assessment" [8] for RA patients, which although not explicitly stated, would likely include screening and monitoring traditional cardiac risks, such as hyperlipidemia. Still, a 2011 report cited that only 32% of general practitioners recognized RA as an independent risk factor for CVD [9], despite growing evidence regarding the heightened risk of CVD associated with RA [10,11].

The goal of this study was to examine the impact of RA upon guideline-recommended diabetes care, including biannual A1c and annual low-density lipoprotein (LDL) cholesterol testing, in a cohort of Medicare patients with diabetes. When examining the impact of comorbidity on care for a given chronic condition, some authors predict less preventive care and guideline adherence in patients with greater comorbidity[12], and others predict more care due to increased visits and provider contacts [13]. A recent model by Piette and Kerr shifts this concept, arguing that rather than the amount of comorbidity, it is the nature of the specific comorbidities and their relationship to the guideline-based tasks that predicts care delivery [14,15]. The model predicts higher quality when the goals of care for two conditions, such as RA and diabetes, align or are "concordant", and lower quality when the goals of care for two conditions are "discordant." We hypothesized that providers would consider glycemic control unrelated to the management of RA (goals are discordant), and that active, symptomatic RA disease would in turn lessen attention to diabetes A1c testing. This predicts decreased A1c testing in diabetes patients when RA is present. In contrast, given the heightened cardiovascular risks associated with both RA and diabetes, we hypothesized that lipid management would be a "concordant" preventive goal in patients with both conditions, predicting increased lipid testing in diabetes patients when RA was also present.

## Materials and methods

### Setting and participants

In this retrospective cohort study, beneficiaries aged 65 and older with diabetes who were continuously enrolled and alive from 2004 to 2006 were identified from a 5% random US Medicare sample, the Medicare Chronic Condition Warehouse dataset [16][17]. Patients were determined to have diabetes using a validated algorithm requiring at least one inpatient or skilled nursing facility claim or more than one professional service claim in 24 months for the following International Classification of Diseases, 9^th ^Revision (ICD-9) codes: 250.xx, 357.2, 362.0x, 366.41, or 648.0x [18].

### Study design

**Figure 1:**
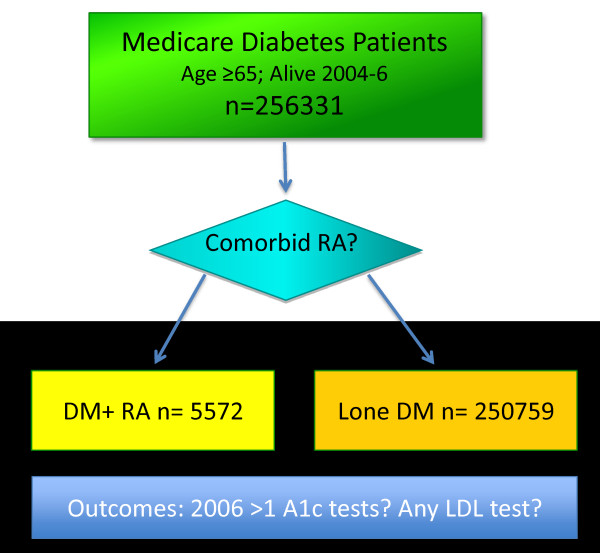
**Cohort selection flow diagram for Medicare diabetes patients with and without comorbid RA**.

Among this cohort of Medicare beneficiaries with diabetes, patients were classified as having or not having RA (see Figure 1) based on the presence or absence of two or more RA ICD-9 codes (714.0 to 714.33) on inpatient or outpatient claims at least 2 months apart in 24 months, using modifications of a previously validated algorithm [19,20]. Both the diabetes and RA diagnostic definitions were applied to the 2004 to 2005 period to establish these diagnoses prior to assessment of 2006 diabetes care measures.

Enrollment and claims data (2004 to 2006) were extracted for all diabetes patients. The Medicare denominator file was used to exclude beneficiaries without continuous Medicare Part A or B coverage or those enrolled in a Medicare health maintenance organization or railroad benefits. We also excluded patients without any outpatient encounters during 2004 to 2006 given the limited opportunity for disease monitoring in such cases. Deaths were captured from the Medicare demographic and enrollment file. The Institutional Review Board at the University of Wisconsin approved this study with a waiver of consent.

Based upon the validated diabetes diagnosis algorithm indicating eligibility for diabetes care metrics in 2006, we identified 256,331 patients with diabetes. Among them, we identified 5,572 (2.2%) older adults with comorbid RA and diabetes.

### Variables

All variables were obtained from Medicare data. Dependent variables included receiving lipid testing (that is, LDL cholesterol testing) and at least two A1c tests in 2006 as assessed by searching carrier, outpatient or inpatient facility claims per prior reports [21,22]. The main explanatory variable was the presence or absence of RA among patients with diabetes.

The Chronic Condition Warehouse contains flags created using numerous established algorithms applied biannually since 1999 to define 21 chronic diseases [16,23-27]. The Chronic Condition Warehouse 2005 end-of-year indicators were used to denote baseline comorbidities, including pre-existing chronic kidney disease, CVD, ischemic heart disease, myocardial infarction (MI), congestive heart failure (CHF) or stroke/transient ischemic attack (TIA) [23-26]. Baseline hyperlipidemia was identified by the presence of more than one ICD-9 code for the condition (272.0 to 272.4) in 24 months from 2004 to 2005 [25,28]. Baseline diabetes complications were defined using a validated algorithm to search for claims through 2005 indicating the presence of lower extremity ulcers, amputation, diabetic eye disease including macular edema or retinopathy, and peripheral vascular disease [29].

Individual sociodemographic and clinical characteristics were included as other potential explanatory variables. These included baseline (2004) age, sex, race, designation of ever receiving Medicaid and zip code residence grouping using US Department of Agriculture census-based Rural Urban Commuting Area (RUCA) codes (urban, suburban, large town or small town based upon population census and commuting flows) [30]. Claims for a gait-assistance device or orthopedic surgery [31,32] during the study period were used to assess musculoskeletal health in the absence of validated administrative indicators of RA severity (code lists available upon request).

Patient risk adjustment was addressed using the Centers for Medicare and Medicaid Services - Hierarchical Condition Categories (HCC) community risk score [33]. The HCC approach creates a score for the beneficiary predicting Medicare expenditures in the subsequent year based on 3,000 ICD-9 codes gathered from all inpatient and outpatient encounters from the prior year (2005) to assign the beneficiary to 70 specific "condition categories", which are clinically and cost similar. In HCC scoring comorbidity is reflected by allowing a beneficiary to belong to multiple condition categories. Beneficiary demographic adjusters are also included (that is, age, gender, Medicaid status, disabled status) in the creation of the final HCC score (average score = 1.0, with higher scores indicating greater risk/complexity).

Measures of utilization included total number of primary care visits each year and total number of unique providers from 2004 to 2006, as well as ever being hospitalized in 2004 to 2006. We used billing dates within the carrier file to determine the number of visits. Provider specialty codes were used to count unique provider totals and distinguish primary care, rheumatology and non-rheumatology specialist visits. Primary care provider (PCP) visits were defined as outpatient encounters with family medicine or internal medicine physicians, nurse practitioners or physician assistants [34,35]. Visits and provider counts with rheumatology and other (non-rheumatology) specialists were also assessed.

### Statistical analysis

Logistic regression with robust estimates of the variance was used to analyze the relationship between explanatory variables and receiving at least two A1c tests or one LDL test. Next, predicted probabilities, adjusted risk ratios (ARR), and odds ratios (OR) for testing were calculated for those with and without comorbid RA . Age, gender, race/ethnicity, Medicaid buy-in, RUCA category, HCC quartile, hospitalization during 2004 to 2006, and other specific comorbidities, including diabetic complications, hyperlipidemia, chronic kidney disease, CVD, orthopedic surgery, gait-assistance device, total number of unique providers seen by quartile and visit frequencies, also were included within logistic models based upon theoretical importance.

Analyses were conducted using SAS version 9.1.3 (SAS Institute, Inc., Cary, NC, USA) and Stata version 10.0 (StataCorp, College Station, TX, USA). Adjusted predicted probabilities were estimated based on the recycled predictions approach using the Stata margins command which facilitated ARR calculation. ARR comparison was included given methodological benefits of this approach when interpreting common outcomes like lipid testing [36]. The recycled predictions approach predicts the outcome (testing) assuming that everyone in the dataset was treated as if they first did and then did not have RA. Confidence intervals were calculated using the delta method and allowed correlation among observations (analogous to the robust option) to estimate the logistic regression [37].

We conducted a sensitivity analysis examining the effect of RA on patients' receipt of at least one instead of two A1c tests. There was no change in directionality, statistical significance or relative magnitude of the main results, and thus we present the original models described above.

## Results

### Descriptive characteristics

Table 1 shows characteristics of our final sample of 256,331 Medicare beneficiaries with diabetes by RA status. In total, 5,572 (2.2%) of these patients had comorbid RA (Table 1). Compared to diabetes patients without RA, those with RA were more likely to be female, receive Medicaid and live in an urban area. On average, diabetes patients with comorbid RA saw more total unique providers over the three-year study period (8.8 ± 5.7 versus 6.3 ± 4.0, *P *<0.01). Similarly, they had more outpatient provider visits per year (14.7 ± 9.3 versus 9.6 ± 6.7, *P *<0.01), although rheumatology encounters represented only on average two (± 3.1) of these visits. Diabetes patients with RA had higher HCC scores (1.55 ± 1.04 versus 0.82 ± 0.80, *P *<0.01) and were more likely to be hospitalized or have orthopedic surgery or gait device history. Patients with comorbid RA also had more CVD, including 28% more CHF, 15% more ischemic heart disease and 11% higher stroke/TIA, but did not differ in rates of baseline MI (*P *= 0.2).

**Table 1:** Characteristics of Medicare diabetes patients with and without RA (N = 256,331)

Characteristic		DM + RA (n = 5,572)	DM no RA (n = 250,759)	*P*-value
**Sociodemographics, n (%) **				
Age	65 to 74 years	3,056 (54.9)	136,288 (54.4)	0.47
	75 to 84 years	2,124 (38.1)	95,540 (38.1)	1
	85+ years	392 (7.0)	18,931 (7.6)	0.16
Female		4,164 (74.7)	151,846 (60.6)	<0.01
Race/ethnicity	White	4,318 (77.5)	207,565 (82.8)	<0.01
	Black	716 (12.9)	26,495 (10.6)	<0.01
	Other	538 (9.7)	16,699 (6.7)	<0.01
Medicaid		1,480 (26.6)	46,944 (18.7)	<0.01
RUCA category	Urban	3,936 (71.8)	164,742 (66.4)	<0.01
	Suburban	407 (7.4)	23,007 (9.3)	<0.01
	Large town	554 (10.1)	31,229 (12.6)	<0.01
	Small town	586 (10.7)	29,033 (11.7)	<0.01
**Baseline cardiovascular profile, n (%)**				
Myocardial infarction		266 (4.8)	11,146 (4.4)	0.24
Ischemic heart disease		3,237 (58.0)	126,690 (50.5)	<0.01
Congestive heart failure		2,063 (37.0)	73,057 (29.1)	<0.01
Stroke/Transient ischemic attack		793 (14.2)	31,971 (12.8)	<0.01
Hyperlipidemia		4,151 (74.5)	190,713 (76.1)	<0.01
**Baseline diabetes complication profile, n (%)**				
Peripheral vascular disease		2,410 (43.3)	82,440 (32.9)	<0.01
Chronic kidney disease		952 (17.1)	35,984 (14.4)	<0.01
Lower extremity ulcers		703 (12.6)	17,815 (7.1)	<0.01
Amputation		50 (0.9)	1,587 (0.6)	<0.05
Diabetic eye disease		765 (13.7)	40,417 (16.1)	<0.01
**Other comorbidity and baseline utilization measures**				
Orthopedic surgery, n (%)		1,452 (26.1)	32,121 (12.8)	<0.01
Gait device, n (%)		492 (8.8)	12,768 (5.1)	<0.01
HCC score, mean (SD)		1.55 (1.04)	0.82 (0.80)	<0.01
Hospitalization 2004 to 2006, n (%)		3,379 (60.6)	125,168 (49.9)	<0.01
Total unique providers, three-year mean (SD)		8.8 (5.7)	6.3 (4.0)	<0.01
Total outpatient visits, annual mean (SD)		14.7 (9.3)	9.6 (6.7)	<0.01
	PCP visits, annual mean (SD)	7.0 (5.9)	5.3 (4.0)	<0.01
	Rheumatology visits, annual mean (SD)	2.0 (3.1)	0.1 (0.5)	<0.01
	Other specialty visits, annual mean (SD)	5.6 (5.8)	4.1 (4.7)	<0.01

DM, Diabetes mellitus; HCC, Hierarchical Condition Categories; PCP, Primary care provider; RA, Rheumatoid arthritis; RUCA, Rural Urban Commuting Area; SD, Standard deviation

### Effect of RA on baseline diabetes complications

Surprisingly, patients with RA differed from patients without RA with regard to four of five baseline diabetes complications. Specifically, diabetes patients with RA were 19% more likely to have chronic kidney disease (unadjusted rate 17.1% compared to 14.4%, *P *<0.01). Lower extremity ulcers were 77% more likely in diabetes patients with RA (13% compared to 7%, *P *<0.01). Peripheral vascular disease was 32% more prevalent among diabetes patients with RA than those without (43% compared to 33%, *P *<0.01), as were amputations (0.9% versus 0.6%, *P *= 0.05). Diabetic eye disease was the only complication observed less frequently in RA (14% compared to 16%, *P *<0.01).

### Diabetes care quality testing performance

#### Effect of RA

The presence of RA among diabetes patients decreased the probability of A1c testing, but slightly increased lipid testing, even after controlling for baseline sociodemographic, comorbidity and utilization differences (Table 2). Before adjustment, 52% of patients with both diabetes and RA versus 57% without RA received at least two A1c tests. After adjustment, diabetes patients with RA were significantly less likely than those without RA to receive the recommended two A1c tests per year (OR 0.84, CI 0.80 to 0.89). Unadjusted LDL testing was similar in both groups (76% with RA versus 77% without), although adjusted models showed that diabetes patients with RA were slightly more likely to be tested (OR 1.08, 1.01 to 1.16).

**Table 2:** Adjusted predicted probabilities and risk ratios for relationship between RA and diabetes testing (N = 256,331)*

	Unadjusted testing (%)	Adjusted predicted probability (%)	95% CI	Odds ratio	95% CI
**Receipt of ≥2 A1c test **					
DM no RA	56.9	57.1	56.9 to 57.3	Referent	
DM + RA	51.8	53.2	51.8 to 54.5	0.84	0.80 to 0.89
**Receipt of ≥1 LDL test **					
DM no RA	76.7	76.7	76.5 to 76.8	Referent	
DM + RA	75.5	77.8	76.8 to 78.8	1.08	1.01 to 1.16

*Models also adjusted for age, gender, race/ethnicity, Medicaid buy-in, RUCA code, HCC quartile, hospitalization in a three-year period, specific co-morbidities including diabetes complications, hyperlipidemia, chronic kidney disease, CVD, orthopedic surgeries, gait device, PCP visits and total providers.

#### Effects of other comorbidities on testing

**Figure 2:**
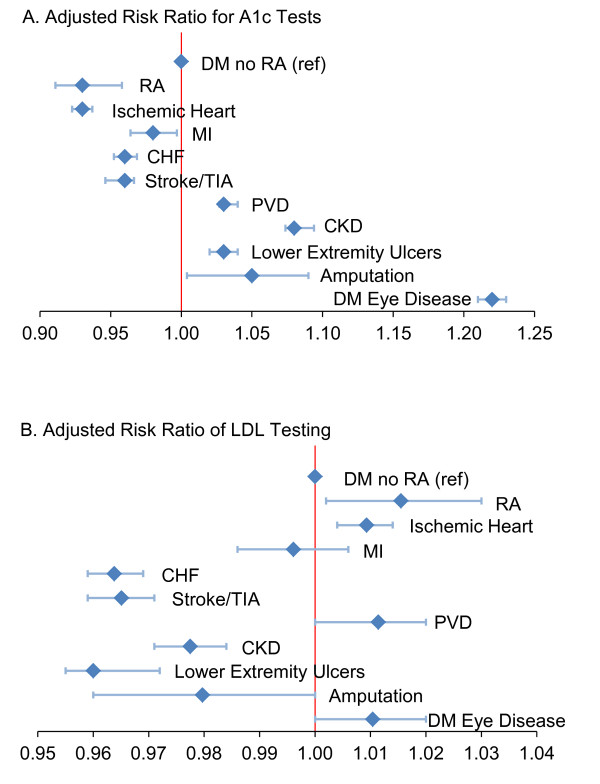
**Multivariate adjusted risk ratios for diabetes testing by additional disease covariates (N=256,331)**. Note that the full model also included age, gender, race/ethnicity, Medicaid buy-in, gait device use, orthopedic surgery status, HCC quartiles, hyperlipidemia, hospitalization status, PCP vists, provider number quartile, and RUCA rurality codes.

Our adjusted model revealed that younger age, female gender and Caucasian race were all significantly associated with receiving recommended diabetes testing (data not shown, see Additional file 1). In addition, the presence of baseline microvascular complications (Figure 2, panel A) was associated with receiving recommended A1c testing (for chronic kidney disease (ARR 1.08 (95% CI 1.07 to 1.09); for lower extremity ulcers ARR 1.03 (1.02 to 1.04); for amputation ARR 1.05 (1.00 to 1.09); for diabetic eye disease ARR 1.22 (1.21 to 1.23); for peripheral vascular disease ARR 1.03 (1.03 to 1.04)). Prevalent CVD burden was associated with less diabetes testing (Figure 2), although acute MI, ischemic heart disease and peripheral vascular and eye disease predicted equivalent or more lipid testing. As expected, measures of worse overall health (hospitalization and highest quartile HCC) were associated with less guideline-recommended diabetes screening, and ambulatory difficulty reflected by use of a gait-assistance device was associated with less diabetes screening (results not shown, see Additional file 1).

## Discussion

In this large national cohort study of Medicare diabetes patients with and without RA, the comorbid presence of RA predicted lower rates of A1c testing, but slightly improved lipid testing, and was associated with higher baseline CVD and diabetes microvascular complications. These findings were consistent with our hypotheses drawn from the Piette and Kerr model theorizing that RA (a condition whose care is discordant from routine diabetes care) would predict less A1c testing. A1c testing is generally perceived as a diabetes care goal but not a RA priority, which might explain lower testing rates in patients who also have competing RA care needs. Lower A1c monitoring in diabetes patients with RA is a problem because poor glycemic control increases the same microvascular complications [5,7] that were more prevalent in patients with RA.

In contrast, diabetes patients with RA were slightly more likely to receive LDL testing. Dual indications for lipid testing in those with both RA and diabetes accurately predicted a concordant motivation for atherosclerosis prevention via LDL testing, per Piette and Kerr. Heightened LDL testing contrasts with our groups' prior reports of low lipid testing in RA patients [38,39], most likely reflecting that in the present study patients were selected for active diabetes, a disease in which lipid performance is routinely monitored and publically reported. The trend for slightly increased lipid testing noted here among diabetes patients with RA also may suggest growing awareness of CVD prevention needs in RA [8].

Prior studies report higher morbidity and mortality among RA patients with diabetes [1,2,40], but to our knowledge no studies have reported heightened prevalence of microvascular complications in patients with comorbid diabetes and RA. We found that CVD, with the exception of MI, was more prevalent in patients with both diabetes and RA. The MI rate is consistent with other RA reports [11], including a Danish study showing similar MI rates in patients with RA, diabetes or both [40]. The more surprising finding was increased chronic kidney disease, lower extremity ulcers and amputations among diabetes patients with RA. While this was an uncontrolled observation outside of our original study question, further investigations to examine RA as a microvascular disease risk seem warranted.

Strengths of this study include the use of a large, nationally representative sample of Medicare patients with diabetes and RA, and extensive demographic, comorbidity and utilization data. However, a few limitations should be noted. First, there is the potential for misclassification of RA and other diagnoses using administrative data. Previously validated algorithms of RA and key conditions were used. Although the strictest validation study used rheumatologist-reported RA coding [19], we adopted the convention of subsequent authors - two or more RA codes in 24 months [20]. Sample definitions might have improved with inclusion of pharmacy data [41], but were not feasible given local data limitations. Results from Medicare patients may also not be generalizable to non-Medicare populations. Lastly, we looked specifically at a 12-month year per guidelines, but performance may have improved with a one-month grace period. Despite these limitations, using this large sample of diabetes patients with and without RA offers new insights.

In light of reduced A1c testing in patients with comorbid RA and diabetes, and heightened microvascular and CVD risk, we urge providers to consider DM and RA as concordant risk factors. We recommend further research examining the interplay between RA and other comorbidities on care quality and outcomes for vascular-risk conditions. This research should examine more direct quality of care measures, including appropriateness of pharmacotherapy and prospective morbidity and mortality. Lastly, given observed lapses in process care measures, such as A1c testing for patients with both RA and diabetes, additional research should investigate optimal co-management or shared disease-management and prevention strategies among specialty and primary care providers.

## Conclusions

Presence of co-morbid RA predicted lower performance of A1c testing among older adults with diabetes. Conversely, receipt of LDL testing was slightly better in patients with RA. Gains for LDL testing might reflect improved recognition of dual testing indications, spurred by EULAR recommendations to perform annual CVD risk assessment and other RA CVD-prevention recommendations. Our findings support conceptualizing RA and diabetes on a concordant risk pathway to improve screening for complications and diabetes monitoring performance in patients with RA. Lower rates of A1c monitoring, in particular, may offer a target for improving the higher-than-expected rates of microvascular complications observed in this study for RA patients with diabetes.

## Abbreviations

ARR: adjusted relative risk; A1c: Hemoglobin A1c; CHF: congestive heart failure; CI: confidence interval; CVD: cardiovascular disease; DM: diabetes mellitus; EULAR: European League Against Rheumatism; HCC: Hierarchical Condition Categories; ICD-9: International Classification of Diseases: 9^th ^Revision; LDL: low-density lipoprotein; MI: myocardial infarction; OR: odds ratio; PCP: primary care physician; RA: rheumatoid arthritis; RUCA: Rural Urban Commuting Area; TIA: transient ischemic attack.

## Competing interests

The authors declare that they have no competing interests.

## Authors' contributions

CB designed the study, performed the statistical analysis and drafted the manuscript. JS participated in statistical analysis and manuscript drafting. CT and AK offered design and analysis consultation and reviewed the final manuscript. NP and KH offered interpretation assistance and critical manuscript revisions. MS facilitated data-acquisition and advised design, analysis and final manuscript review for the study. All authors read and approved the final manuscript.

## Supplementary Material

Additional file 1 **Multivariate adjusted risk ratios for diabetes testing by additional covariates (N = 256,331)**. Multivariate adjusted risk ratios for diabetes testing by additional covariates, including age, sex, race/ethnicity, baseline comorbidities, HCC quartile, hospitalization, orthopedic surgery, annual PCP visits and lowest total provider quartile. Click here for file
