# Supplementary material for: Monitoring diabetes in patients with and without rheumatoid arthritis: a Medicare study
Source: Arthritis Res Ther. 2012 Jul 18;14(4):R166. doi: 10.1186/ar3915 (PMC3580560; doi:10.1186/ar3915)
Supplement: Additional file 1 — Multivariate adjusted risk ratios for diabetes testing by additional covariates (N = 256,331). Multivariate adjusted risk ratios for diabetes testing by additional covariates, including age, sex, race/ethnicity, baseline comorbidities, HCC quartile, hospitalization, orthopedic surgery, annual PCP visits and lowest total provider quartile. [file ar3915-S1.DOCX]

| **Appendix Table. Multivariate adjusted risk ratios for diabetes testing by additional covariates (N=256331)*** | | | | | | | |
| --- | --- | --- | --- | --- | --- | --- | --- |
| **Characteristic** | | **Adjusted  Risk Ratio HbA1c Testing** | **95% CI** | **Adjusted  Risk  Ratio LDL  Testing** | **95% CI** | **Adjusted  Risk  Ratio Eye Exams** | **95% CI** |
| Age: | 65-74 years | (Reference) |  | (Reference) |  | (Reference) |  |
|  | 75-84 years | 0.96 | 0.95-0.97 | 0.97 | 0.96-0.97 | 1.07 | 1.07-1.08 |
|  | 85+ years | 0.89 | 0.87-0.90 | 0.87 | 0.86-0.88 | 1.09 | 1.07-1.10 |
| Female | | 1.06 | 1.05-1.07 | 1.02 | 1.02-1.03 | 1.07 | 1.06-1.08 |
| Race/ethnicity: | White | (Reference) |  | (Reference) |  | (Reference) |  |
|  | Black | 0.96 | 0.95-0.97 | 0.97 | 0.97-0.98 | 0.98 | 0.97-0.99 |
|  | Other | 0.87 | 0.85-0.88 | 0.98 | 0.97-0.99 | 0.94 | 0.92-0.95 |
| *[DATA SHOWN IN FIGURE 1]* | | | | | | | |
| *Baseline RA* | | *0.93* | *0.91-0.95* | *1.02* | *1.00-1.03* | *1.04* | *1.02-1.06* |
| *Baseline ischemic heart disease* | | *0.93* | *0.92-0.94* | *1.01* | *1.00-1.01* | *0.97* | *0.96-0.98* |
| *Baseline MI* | | *0.98* | *0.96-1.00* | *1.00* | *0.99-1.01* | *0.95* | *0.94-0.97* |
| *Baseline CHF* | | *0.96* | *0.95-0.97* | *0.96* | *0.96-0.97* | *0.95* | *0.95-0.96* |
| *Baseline stroke/TIA* | | *0.96* | *0.95-0.97* | *0.97* | *0.96-0.97* | *0.97* | *0.96-0.98* |
| *Baseline hyperlipidemia* | | *1.30* | *1.29-1.31* | *1.42* | *1.41-1.43* | *1.07* | *1.06-1.08* |
| *Baseline peripheral vascular disease* | | *1.03* | *1.03-1.04* | *1.01* | *1.00-1.02* | *1.02* | *1.01-1.02* |
| *Baseline chronic kidney disease* | | *1.08* | *1.07-1.09* | *0.98* | *0.97-0.98* | *0.97* | *0.96-0.98* |
| *Baseline lower extremity ulcers* | | *1.03* | *1.02-1.04* | *0.96* | *0.96-0.97* | *0.97* | *0.96-0.98* |
| *Baseline amputation* | | *1.05* | *1.00-1.09* | *0.98* | *0.96-1.00* | *0.89* | *0.86-0.93* |
| *Baseline eye disease* | | *1.22* | *1.21-1.23* | *1.01* | *1.00-1.02* | *1.33* | *1.32-1.34* |
| Highest HCC quartile | | 0.98 | 0.97-0.99 | 0.93 | 0.93-0.94 | 0.96 | 0.95-0.97 |
| Hospitalization (ever) | | 0.93 | 0.92-0.94 | 0.94 | 0.94-0.95 | 0.90 | 0.89-0.90 |
| Orthopedic surgery (ever) | | 0.98 | 0.97-0.99 | 1.00 | 1.00-1.01 | 1.03 | 1.02-1.03 |
| Annual PCP visits: | <2/year | Referent |  | Referent |  | Referent |  |
|  | ≥2/year | 1.24 | 1.22-1.25 | 1.11 | 1.11-1.11 | 1.08 | 1.07-1.09 |
| Lowest total provider quartile | | Referent |  | Referent |  | Referent |  |
|  | Second quartile | 1.06 | 1.05-1.07 | 1.06 | 1.05-1.06 | 1.27 | 1.26-1.28 |
|  | Third quartile | 1.09 | 1.08-1.10 | 1.07 | 1.06-1.08 | 1.42 | 1.41-1.44 |
|  | Highest quartile | 1.10 | 1.09-1.11 | 1.10 | 1.10-1.11 | 1.59 | 1.57-1.60 |
| *Models also adjusted for age, gender, race/ethnicity, Medicaid buy-in, PCP visits, provider number quartile, and RUCA rurality codes. RA=Rheumatoid arthritis; LDL=Low-density lipoprotein cholesterol; MI=Myocardial infarction; CHF=Congestive heart failure; TIA=Transient ischemic attack; HCC=Hierarchical Condition Categories scale; RUCA=Rural Urban Commuting Area; PCP=Primary care provider | | | | | | | |
